# Analysis of gut microbiota with cryptosporidiosis based on fecal condition in neonatal dairy calves on a farm in Japan

**DOI:** 10.3168/jdsc.2023-0539

**Published:** 2024-05-10

**Authors:** Yasuhiro Morita, Momoko Yachida, Keita Tokimitsu, Megumi Itoh

**Affiliations:** Division of Clinical Veterinary Medicine, Department of Veterinary Medicine, Obihiro University of Agriculture and Veterinary Medicine, Hokkaido, 080-8555 Japan

## Abstract

Cryptosporidiosis is a major cause of diarrhea and is associated with high morbidity in calves. Changes in the gut microbiota exacerbate diarrhea caused by *Cryptosporidium parvum* infection in neonatal and weaned calves. However, information on the gut microbiota of neonatal calves with *C. parvum* infection is scarce, and research into the microbiome of calves is essential for developing preventive and therapeutic interventions. This study aimed to elucidate the gut microbiota of neonatal calves with cryptosporidiosis. We collected 31 fecal samples from 31 neonatal calves on a dairy farm with or without *C. parvum* antigen [CP(+) or CP(−)] using a kit and analyzed the differences in the microbiota between diarrheal (D) and normal (N) fecal samples with *C. parvum* infection based on the fecal score. The analyses revealed the α diversity indexes of fecal microbiota in CP(+)-N samples were higher than that in CP(+)-D samples. *Megasphaera* spp. and other rumen microbes were identified, and significantly associated with CP(+)-N samples compared with CP(+)-D samples by linear discriminant analysis effect size (LEfSe). We conclude that the specific gut microbiota could characterize fecal microbiota in calves with neonatal cryptosporidiosis without clinical symptoms.

## Highlights

• Fecal microbiota in nondiarrheal *C. parvum*-positive calves had a higher alpha diversity.
• Microbes found in nondiarrheal calves with cryptosporidiosis have the potential to prevent cryptosporidiosis.
• Megasphaera spp. and other rumen-related microbes were found in normal fecal samples with *C. parvum* antigen.

## Graphical Abstract

**Figure:**
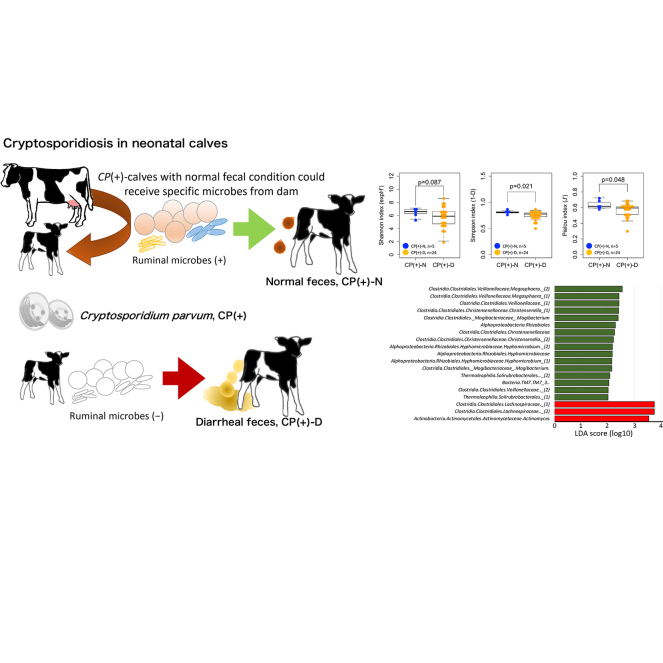
**Summary:** Cryptosporidiosis is a major cause of diarrhea and is associated with high morbidity in calves. This study aimed to elucidate the gut microbiota of neonatal calves with cryptosporidiosis. We collected 31 fecal samples from 31 neonatal calves with or without *Cryptosporidium parvum* antigen and analyzed the differences in microbiota between diarrheal and normal fecal samples with *C. parvum* infection based on the fecal score. The analyses revealed a higher alpha diversity of fecal microbiota in *C. parvum*-positive calves that did not exhibit diarrhea than in calves that exhibited diarrhea. Megasphaera spp. and other rumen-related microbes were specifically found in normal fecal samples with *C. parvum* antigen, and we conclude that these microbes are present in neonatal cryptosporidiosis calves without clinical symptoms.

Cryptosporidiosis, a major cause of neonatal diarrhea, has high morbidity in calves (Wyatt et al., 2010) and generally infects preweaning calves less than 6 wk of age (Thomson et al., 2019). Cryptosporidiosis is caused by *Cryptosporidium* spp., and *C. parvum* is one of the main gastroenteric pathogens. Calves are mainly infected with *C. parvum* via the fecal-oral route (McDonald, 2000). Infections have a significant impact on the productivity and economy of farms in terms of milk and meat production, as they reduce growth rates and can cause death (Brook et al., 2008). Prevalence of *C. parvum* in fecal samples of cattle herds was reported to range from 13% to 100% in Europe (Imre and Dărăbuș, 2011) and was also high at 1.7% to 100% in Japan (El-Alfy and Nishikawa, 2020). A previous study indicated a correlation between parasite burden and diarrhea severity (Operario et al., 2015); however, another study indicated that infected calves show no or mild symptoms (Shaw et al., 2020). This inconsistency could be a result of the host intestinal microbiota and immune system (El-Alfy and Nishikawa, 2020; El-Deeb et al., 2022).

An association exists between the presence of *Cryptosporidium* spp. and intestinal microbiota, including a significant increase in *Fusobacterium* spp. in the feces of calves with cryptosporidiosis (Dorbek-Kolin et al., 2022). The onset of diarrhea in calves may be associated with the gut microbiota. Chen et al. (2022) suggested that interactions among some bacteria could influence calf diarrhea and that some species of *Prevotella* spp. may be the core microbiota in calves. These results could indicate that changes in the gut microbiota are also one of the exacerbating factors in diarrhea caused by *Cryptosporidium* spp. infection.

However, information on the gut microbiota in neonatal calves with *Cryptosporidium* spp. infection without diarrheal symptoms is scarce. Information from *Cryptosporidium* spp. infection-positive with nondiarrheal fecal samples is needed to develop preventive and therapeutic interventions. Therefore, this study aimed to elucidate the characteristics of the gut microbiota of neonatal calves with cryptosporidiosis based on clinical symptoms, on a dairy farm.

The study was conducted from June to November 2022 on a dairy farm (300 lactating cows) in Japan (42°45′36ʺN, 143°03′00ʺE). Thirty-one dairy calves (Holstein-Friesian, Jersey, and Japanese Black cattle [by embryo transfer] until 1 to 2 wk old) were randomly selected and assigned in this study. The calves were housed in wooden pens individually and received the same feeding management after dam-calf separation. Dam-calf separation was performed approximately 12 h after birth with calves receiving colostrum of the dam with feeding bottles, estimated to contain sufficient concentrations of IgG using a digital Brix refractometer instrument referring to Bielmann et al. (2010). If the IgG levels in the colostrum of the dam were low, the calf was fed powdered colostrum (225 g, HeadStart, Saskatoon Colostrum Company Ltd., Saskatoon, SK, Canada) twice on the day of birth. Fecal samples were collected from the calves during their scheduled health examination the week after birth (1–2 wk of age). Fecal *Cryptosporidium parvum* antigen was detected using a kit (DipFit *Cryptosporidium parvum*, BIO K387, Bio-X Diagnostics S.A., Rochefort, Belgium) and the presence of the *Cryptosporidium* spp. antigen was recorded. Fecal samples were transported on ice to the laboratory and stored at −30°C for less than 6 mo until transportation for analysis. The fecal condition was evaluated on the farm by 2 veterinarians in our hospital, using fecal consistency scoring (**FCS**), as described by Renaud et al. (2020): 0 = normal (firm but not hard); 1 = soft (does not hold form, piles, but spreads slightly); 2 = runny (spreads readily); and 3 = watery (liquid consistency, splatters). A score of ≥2 indicated the presence of diarrhea. All experimental procedures were approved by the Committee of the Care and Use of Experimental Animals at Obihiro University and Agriculture and Veterinary Medicine (approval number 22–229).

The 16S rRNA gene is unique to prokaryotes (bacteria and archaea) and is most commonly used for bacterial species identification because of its extensive sequence databases. Therefore, a 16S rRNA library was used in this study. Gene extraction from samples, library preparation, and identification of bacterial species using the 16S rRNA gene database of bacterial reference strains were performed by Seibutsu Giken Co. (Sagamihara, Kanagawa, Japan). Briefly, fecal samples were freeze-dried and homogenized, and the supernatant was separated for DNA extraction. The library was prepared from the purified samples using a 2-step tailed PCR method. The DNA was amplified via PCR using the following primers, according to Klindworth et al. (2013). The bacterial 16S rRNA sequence of the V3–V4 region was amplified using 2 sets of primers; first-round: V3–V4f_MIX (5ʹ-ACACTCTTTCCCTACACGACGCTCTTCCGATCT-NNNNN-CCTACGGGNGGCWGCAG-3ʹ) and V3-V4r_MIX (5ʹ-GTGACTGGAGTTCAGACGTGTGCTCTTCCGATCT-NNNNN-GACTACHVGGGTATCTAATCC-3ʹ), and second-round: 2ndF(5′-AATGATACGGCGACCACCGAGATCTACAC-Index2-ACACTCTTTCCCTACACGACGC-3′) and 2ndR (5′-CAAGCAGAAGACGGCATACGAGAT-Index1-GTGACTGGAGTTCAGACGTGTG-3′). Sequencing was performed using the MiSeq system with MiSeq Reagent Kit v3 (Illumina, San Diego, CA) at 2 × 300 bp. The Fastq_barcode_splitter from the Fastx toolkit (ver. 0.0.14) was employed to selectively extract sequences that exactly matched the primers. Sequences with a low-quality score (<20) were removed, resulting in a final length of 130 bp or less. Preprocessed sequences were then analyzed using Quantitative Insights into Microbiology Ecology (QIIME) 2 (v2022.8) and clustered into operational taxonomic units (**OTU**) based on the Greengenes database (https://greengenes.lbl.gov/Download/), with a 97% similarity threshold.

Alpha diversity metrics using the Shannon index (diversity, “shannon,” expH'), Simpson index (diversity, “simpson”), and Pielou index (evenness) were calculated to detect differences between *C. parvum* antigen-positive [**CP(+)**] and antigen-negative [**CP(−)**] groups and between fecal samples from calves with and without diarrhea (**N**: samples with normal fecal condition, **D**: samples with diarrheal condition, based on the scoring) in the CP(+) groups. Statistical significance among groups in α diversity metrics was analyzed using Welch's *t*-test. Beta diversity was assessed using principal component analysis (**PCA**, prcomp) of the robust Aitchison distances (vegdist, “robust.aitchison”) in each fecal sample from each group (Martino et al., 2019). To determine the microbiome variation attributable to individual samples, a permutational ANOVA (PERMANOVA, adonis2) was performed with permutations = 100,000. These analyses were conducted using R software version 4.3.2 (http://www.R-project.org/), with the “vegan” and “stats” packages; the results were deemed statistically significant at *P* ≤ 0.05.

The linear discriminant analysis effect size (**LEfSe**) approach was used to identify microbial taxa that were significantly associated with the groups, according to Segata et al. (2011). Briefly, the LEfSe algorithm contains a Kruskal–Wallis rank sum test to detect differences between classes and linear discriminant analysis (**LDA**) to detect differences in the relevant features. The parameters were set at *P* = 0.05 and LDA score = 2.0 for computation. The LEfSe was performed using the LEfSe Docker container of the biobakery account (biobakery/LEfSe v1.0.0): the dataset table was converted into LEfSe format (format_input.py), and LEfSe was executed (run_lefse.py) with the specified settings and no subclass specifications.

At the time of the first fecal examination in the neonatal area of the farm, most calves tested positive for *C. parvum* antigens (29/31, 93.5%). Five of the 29 CP(+) fecal samples (17.2%) had normal fecal characteristics [**CP(+)-N**, FCS = 0 and 1], and 24 (82.8%) had runny-to-watery diarrhea [**CP(+)-D**, FCS = 2 and 3]. In the follow-up survey conducted 3 mo after sampling, it was noted that 17 of the 29 CP(+) calves required medical treatments, whereas 12 calves maintained their vigor and appetite. No significant difference was observed in the frequency of treatments between CP(+)-N and CP(+)-D calves. Figure 1 shows the relative abundance of fecal microbes in CP(+) and CP(−) calves in phylum (A) and family (B) levels. There was no significant difference in the α and β diversity of fecal microbiota between CP(+) and CP(−) calves. Differences in the microbiota of CP(+)-N and CP(+)-D calves are shown in Figure 2. The α-diversity of the fecal microbiota in CP(+)-N samples was higher than that in CP(+)-D samples, as evidenced by the Simpson index (Figure 2B) and Pielou index (Figure 2C). Moreover, we observed a tendency for higher values in the Shannon index calculations (Figure 2A). This study revealed a high diversity of fecal microbiota in CP(+)-N. The β-diversity between CP(+)-N and CP(+)-D showed no significant difference (PERMANOVA; *P* = 0.8832, Figure 2D). In contrast, in the LEfSe analysis, 19 microbes were identified in CP(+)-N (n = 16) and CP(+)-D (n = 3). Specifically, *Megasphaera* spp., *Christensenella* spp., *Mogibacterium* spp., and *Saccharibacteria* (*TM7*) spp. were identified in CP(+)-N samples, whereas *Lachnospiraceae* and *Actinomycetaceae* were identified in CP(+)-D samples (Figure 2E, F).

**Figure 1:**
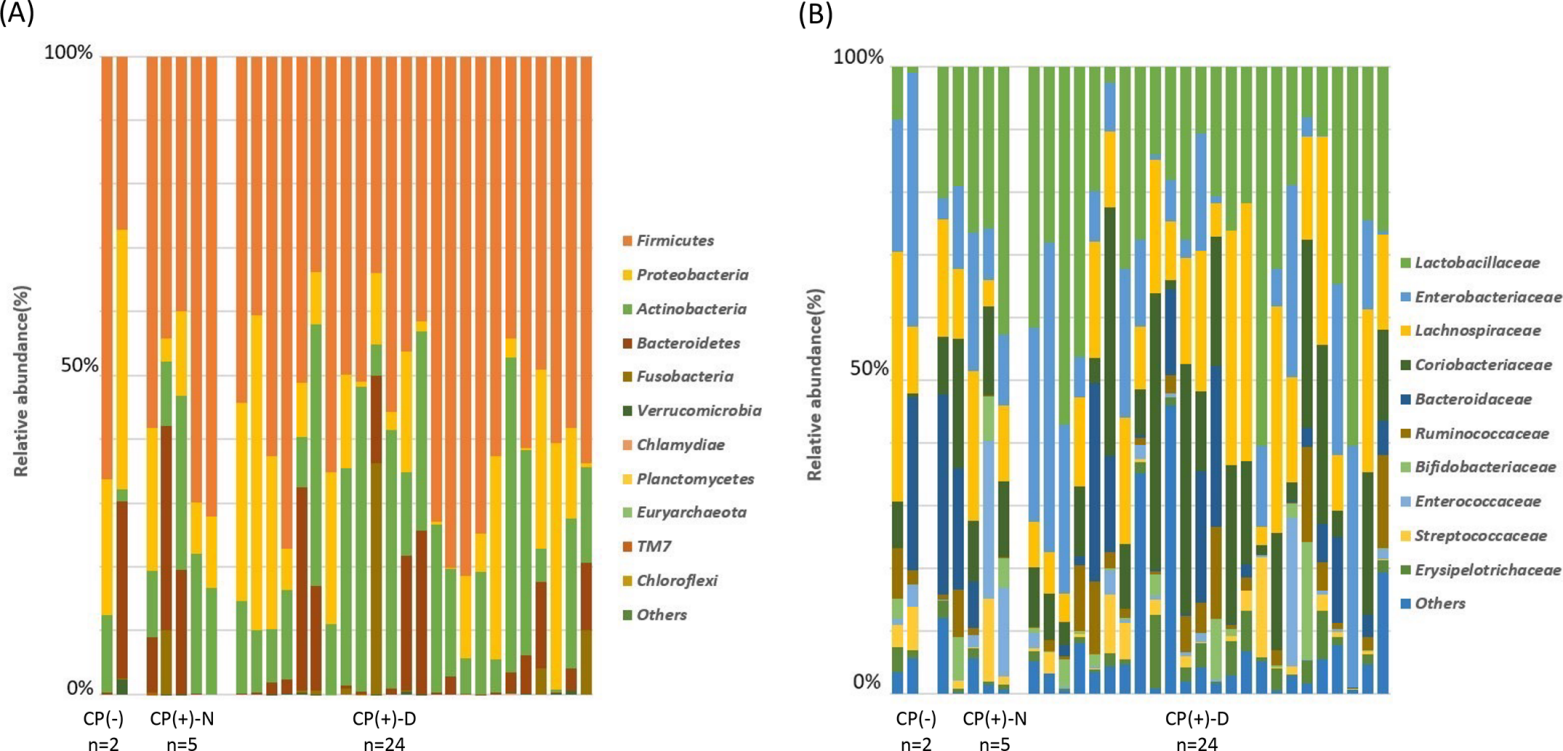
Microbial composition at the phylum (A) and family (B) levels. *Cryptosporidium* infection negative (−; n = 2) and positive (+; n = 29), and *Cryptosporidium* spp. positive infection with normal feces [CP(+)-N; n = 5] and with diarrhea [CP(+)-D; n = 24].

**Figure 2:**
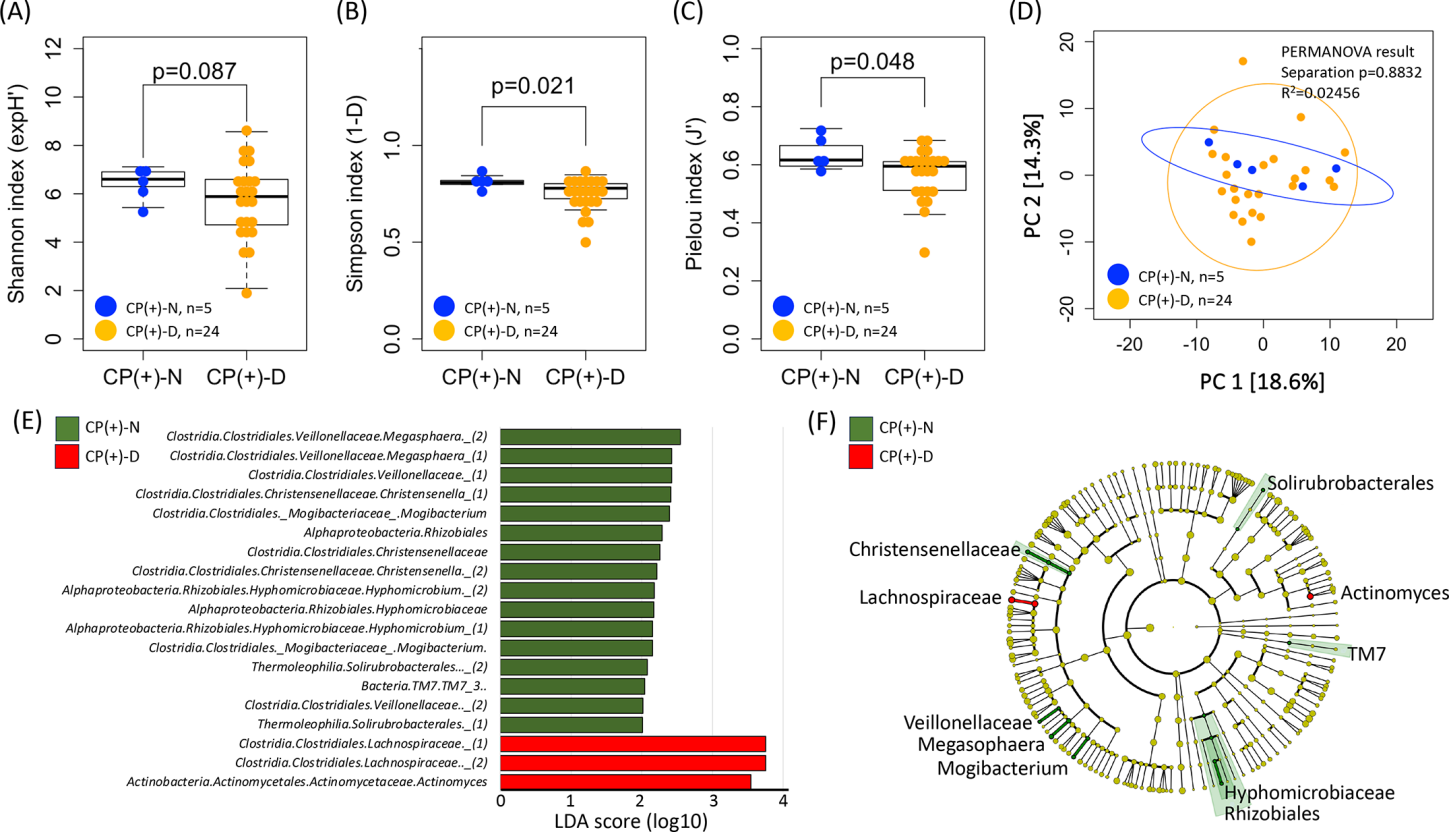
Summary of α and β diversity of fecal microbiota with cryptosporidiosis. The blue and orange circles indicate calves with cryptosporidiosis showing nondiarrheal [CP(+)-N] and diarrheal [CP(+)-D] symptoms, respectively. Alpha diversity index based on the Shannon (A), Simpson (B), and Pielou (C) indices. The thick line in the middle of the box indicates the median value. The top and bottom of the box represent the third and the first quartile, respectively. The upper and lower whiskers indicate the largest and smallest data points, respectively. Beta diversity was assessed using principal component analysis (PCA) of robust Aitchison distances within each fecal sample from each group based on the bacterial 16S rRNA gene sequence data for fecal samples (D). Permutational multivariate ANOVA (PERMANOVA) clustering and differences in dispersion results are indicated, along with a 95% CI. Linear discriminant analysis (LDA) scores of abundant taxa (E) by LEfSe analysis and cladogram (F) in the *Cryptosporidium*-infected but nondiarrhea feces. The red and green highlighted objects indicate specific microbes in the samples of calves with cryptosporidiosis showing diarrheal symptoms and calves with cryptosporidiosis showing nondiarrheal symptoms, respectively.

*Megasphaera* spp. synthesize short-chain fatty acids (Yoshikawa et al., 2018) as lactic acid bacteria, and previous studies have suggested the regulation and maintenance of intestinal homeostasis (Parada Venegas et al., 2019), locally regulating the host intestinal immune response in animals (Binder, 2010; Bachem et al., 2019). Additionally, a decrease in short-chain fatty acid levels is associated with an increase in *C. parvum* infection in mice (Charania et al., 2020). Moreover, *Megasphaera elsdenii* is used as a probiotic in cattle to treat metabolic acidosis and improve productivity as a commercial product (Lopes et al., 2021) because it can convert lactate to short-chain fatty acids (Sedighi and Alipour, 2019) and maintain ruminal function (Counotte et al., 1981). Additionally, *Megasphaera* spp. was significantly associated with CP(+)-N samples, and could characterize fecal microbiota in calves with neonatal cryptosporidiosis without clinical symptoms. Consequently, *Megasphaera* spp. may serve as a promising candidate for probiotic and therapeutic interventions against cryptosporidiosis.

*Christensenellaceae*, a recently described family in the phylum *Firmicutes*, is generally detected in mammalian intestine and rumen (Ramayo-Caldas et al., 2020). Previous studies have considered this microbe as an important hydrogen-producing bacterial group used for metabolism in the human intestine (Waters and Ley, 2019; Miura et al., 2021) and methane production in the rumen (Andrade et al., 2022). However, their role in the guts of ruminants, particularly neonatal calves, remains unclear. Additionally, *Mogibacterium* and *Saccharibacteria* (*TM7*) are abundant in rumen fluid. An increased abundance of *Mogibacterium* was associated with high methane production in the rumen (Wallace et al., 2015). Additionally, *Saccharibacteria* may contribute to improved cellulose degradation (Opdahl et al., 2018). Fecal samples were collected from 2-wk-old calves without a fully developed rumen. Therefore, these microbes may have been transferred to the calves from their dams or the environment. Microbial transfer from dams to calves is important for constructing the calf microbiota and influencing their subsequent formation (Yeoman et al., 2018). Therefore, calves that did not present with diarrhea may have received sufficient microbiota from their dams in the farm environment.

*Lachnospiraceae* and *Actinomycetaceae* were specifically identified in diarrheal samples with *C. parvum* in the current study by LEfSe analysis. Consistent with our results, Xin et al. (2021) identified *Actinomycetaceae*, *Enterobacteriaceae*, and *Fusobacteriaceae* as specifically detected in diarrheic calves, suggesting that these differences affected short-chain fatty acid production in the intestine. Conversely, an earlier study indicated that *Lachnospiraceae* was more abundant in fecal samples from diarrheal calves treated with antibiotics (Kim et al., 2021) and can potentially reduce inflammatory response in the intestine (Liu et al., 2022). In the current study, these microbes may be related to the anti-inflammatory response in the intestine.

Here, we could not measure the level of blood immunoglobulins in calves and could only estimate the colostrum intake from the dam using Brix values. Consequently, the evaluation of calves' immune response to *Cryptosporidium* spp. in this study is limited by this constraint. A previous study indicated the level of clinical features associated with the immune response of calves in cryptosporidiosis (El-Alfy and Nishikawa, 2020; El-Deeb et al., 2022). Here, all calves could receive sufficient immunoglobulins either from the dam's colostrum or powdered colostrum, resulting in robust vigor and appetite in all calves before fecal sampling at age 1 to 2 wk. We presumed that all experimental calves received sufficient levels of immunoglobulins, and that the composition of the intestinal microbiota is one of the factors in whether calves infected with *Cryptosporidium* spp. show clinical signs of diarrhea or not.

We focused on nondiarrheal calves infected with *Cryptosporidium* spp., and this is a novel study suggesting that some bacteria could prevent the clinical burden. Summarily, the microbial composition of nondiarrheal calves with cryptosporidiosis could differ from that of calves with diarrhea. Although further analysis of the gut microbiota of animals with cryptosporidiosis showing no diarrheal symptoms is needed, the microbes specifically identified in fecal samples from nondiarrheal calves with cryptosporidiosis, such as *Megasphaera* spp. and other rumen microbes, may have the potential to reduce clinical burden associated with cryptosporidiosis in calves.
